# The Thyroid Hormone Analog GC‐1 Mitigates Acute Lung Injury by Inhibiting M1 Macrophage Polarization

**DOI:** 10.1002/advs.202401931

**Published:** 2024-10-07

**Authors:** Bin Li, Cong Xia, Wanyu He, Jingyi Liu, Ruoyu Duan, Zhihua Ji, Xiaoyue Pan, Yanlin Zhou, Guoying Yu, Lan Wang

**Affiliations:** ^1^ Pingyuan Laboratory State Key Laboratory of Cell Differentiation and Regulation Henan International Joint Laboratory of Pulmonary Fibrosis Henan Center for Outstanding Overseas Scientists of Organ Fibrosis College of Life Science Henan Normal University Xinxiang 453007 P. R. China; ^2^ College of Chemical and Pharmaceutical Engineering Huanghuai University Zhumadian 463000 P. R. China

**Keywords:** ALI/ARDS, DNMT3b‐PPARγ‐NF‐κB pathway, GC‐1, macrophage polarization, thyroid hormone

## Abstract

Acute lung injury (ALI)/acute respiratory distress syndrome (ARDS) is a life‐threatening condition with a high mortality rate of ≈40%. Thyroid hormones (THs) play crucial roles in maintaining homeostasis of the cellular microenvironment under stress. The previous studies confirmed that the clinical‐stage TH analog GC‐1 significantly alleviates pulmonary fibrosis by improving the function of mitochondria in epithelial cells. However, the effects of GC‐1 on macrophages in lung injury and the related mechanisms remain unclear. This study evaluated the therapeutic effects of GC‐1 in two murine models of lipopolysaccharide (LPS)‐ or hydrochloric acid (HCl)‐induced ALI. Additionally, mouse alveolar macrophages (AMs) and human THP‐1‐derived macrophages are utilized to investigate the impact of GC‐1 on macrophage polarization. GC‐1 effectively reduces the inflammatory response and lung injury in ALI mice, as evidenced by neutrophil infiltration, cytokine levels, alveolar fluid clearance, and pulmonary pathology. Notably, GC‐1 selectively inhibits M1 macrophage polarization, which may be achieved by impeding NF‐κB signaling activation through the DNMT3b‐PPARγ‐NF‐κB pathway in a TH receptor β1 (TRβ1)‐dependent manner, consequently suppressing the polarization of macrophages toward the M1 phenotype and overproduction of inflammatory cytokines. Overall, these findings highlight the immunomodulatory property of GC‐1 as an anti‐inflammatory strategy for ALI/ARDS and inflammation‐related diseases.

## Graphical abstract

Current study reveals that TH analog GC‐1 may suppress NF‐κB signaling activation, selectively blocking M1 macrophage polarization through DNMT3b‐PPARγ‐NF‐κB pathway dependent on TRβ1. GC‐1 effectively reduces inflammation and acute lung injury in mice induced by LPS or HCl, might be a promising targeted therapy for ALI/ARDS. This advances the understanding of the role of GC‐1 in lung inflammation and repair.

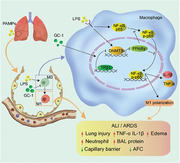

## 1 Introduction

ALI/ARDS is a severe respiratory disorder caused by noncardiogenic factors, leading to acute and progressive hypoxic respiratory failure.^[^ ^1^ ^]^ Due to the absence of direct treatment options, ALI/ARDS is a major cause of death in critically ill patients, with a mortality rate of ≈40%.^[^ ^2^ ^]^ The COVID‐19 outbreak has led to heterogeneity in the pathogenesis of ALI/ARDS,^[^ ^3^ ^]^ which has resulted in the failure of potential treatments such as aspirin^[^ ^4^ ^]^ and vitamin D.^[^ ^5^ ^]^ Precision medicine has revealed different types of ARDS, including direct and indirect forms,^[^ ^6^ ^]^ as well as hyperinflammatory and hypoinflammatory subtypes.^[^ ^7^ ^]^ Increasing evidence suggests that targeting specific molecular characteristics and personalized treatment strategies offer hope for effectively addressing ARDS.

TH is crucial for growth, development, and metabolism.^[^ ^8^ ^]^ Studies have shown that TH can be used to treat hyperlipidemia, obesity, acute myocardial infarction, and cognitive dysfunction.^[^ ^9^ ^]^ GC‐1, also known as Sobetirome, is a synthetic mimetic of T_3_ that selectively binds to the TH receptor β1 without causing cardiac thyrotoxicosis. Encouragingly, phase I clinical trials have demonstrated that GC‐1 is both effective and safe in reducing low‐density lipoprotein cholesterol levels, despite funding constraints preventing the completion of phase II trials.^[^ ^10^ ^]^ Additionally, GC‐1 can be used instead of THs for managing dyslipidemia, hepatocellular carcinoma, and demyelinating diseases.^[^ ^11^ ^]^ More studies have shown that TH can alleviate ALI induced by LPS, ventilators, and sepsis through mechanisms involving an increased alveolar fluid clearance rate,^[^ ^12^ ^]^ maintenance of surfactant homeostasis,^[^ ^13^ ^]^ augmentation of macrophage phagocytosis,^[^ ^14^ ^]^ and a reduction in chemokine and cytokine levels.^[^ ^15^ ^]^ Our previous findings also demonstrated that T_3_ and GC‐1 can restore mitochondrial function in alveolar epithelial cells, effectively reducing pulmonary fibrosis and lung injury in mice.^[^ ^16^ ^]^ These findings strongly indicate that TH and thyromimetics hold promising therapeutic potential for pulmonary diseases.

Macrophages play crucial roles in maintaining homeostasis in the lung microenvironment and exhibit both proinflammatory and anti‐inflammatory functions during ALI/ARDS. Modulating their polarization could be a potential therapeutic approach for treating these conditions.^[^ ^17^ ^]^ There is a growing focus on the immunoregulatory effects of TH. Studies have shown that the T_3_ concentration affects macrophage activity.^[^ ^18^ ^]^ Hyperthyroidism reduces inflammation, whereas hypothyroidism promotes phagocytosis and increases IL‐1β expression in macrophages.^[^ ^19^ ^]^ The effects of TH and thyromimetics on macrophage polarization in ALI/ARDS are still unclear despite some evidence suggesting that T_3_ can reverse proinflammatory cytokine levels in hypothyroid or type 2 deiodinase knockout mice.^[^ ^15^ ^]^

The present study aimed to explore the potential of GC‐1 in reducing lung injury through the modulation of macrophage polarization. The therapeutic effect of GC‐1 was evaluated by assessing lung tissue pathology, neutrophil infiltration, and inflammatory factors in two mouse models. Furthermore, the influence of GC‐1 on macrophage polarization was elucidated by investigating NF‐κB signaling and cytokine levels in mouse alveolar macrophages (AMs) and human THP‐1 macrophages. Additionally, the potential mechanism was explored by examining the role of GC‐1 in the interaction between PPARγ and DNMT3b, as well as the impact of TRβ1 on GC‐1.

## 2 Results

### 2.1 GC‐1 Ameliorated ALI Induced by LPS and HCl in Mice

**Figure 1:**
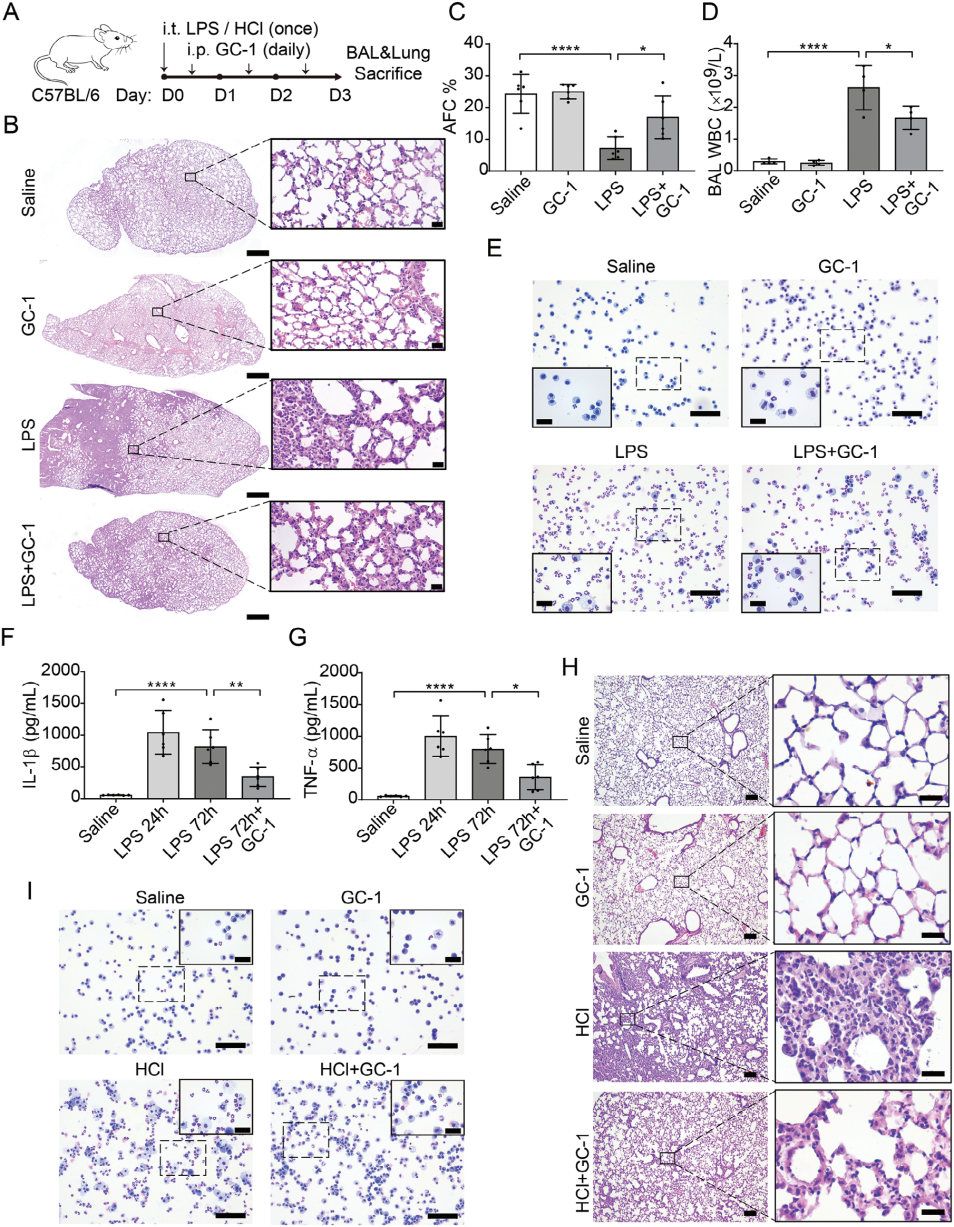
GC‐1 mitigated lung injury, neutrophil infiltration, and proinflammatory cytokine production in mice with LPS‐ or HCl‐induced ALI. A) Schematic representation of the experiment. C57BL/6 mice were challenged intratracheally with LPS (5 mg kg^−1^ body weight) or HCl (0.1 m, pH 1.0) on Day 0 followed by daily intraperitoneal injection of GC‐1 (100 µg kg^−1^ body weight) or saline for 3 days. B) Representative histological sections of LPS‐induced lung tissue stained with H&E (*n* = 3), Scale bars: 500 and 20 µm (insets). LPS‐induced lung injury was evaluated by C) alveolar fluid clearance (*n* = 6), and D) the concentration of white blood cells (WBC) recovered from BAL fluid (*n* = 4). E) Representative LPS‐induced BAL cell smear stained with Diff‐Quik (*n* = 4), Scale bars: 100 and 20 µm (insets). F) IL‐1β and G) TNF‐α levels in LPS‐induced BAL fluid were measured by ELISA (*n* = 6). H) Representative H&E staining of HCl‐induced lung histological sections (*n* = 3), Scale bars: 100 and 20 µm (insets). I) Representative HCl‐induced BAL cell smear stained with Diff‐Quik (*n* = 6), Scale bars: 100 and 20 µm (insets). The values are shown as mean ± SD. ^*^ *p* < 0.05; ^**^ *p* < 0.01; ^****^ *p* < 0.0001.

To evaluate the therapeutic efficacy of GC‐1 in LPS‐induced ALI, we administered a single dose of LPS intratracheally to mice, followed by intraperitoneal injection of GC‐1 (**Figure** **1**A). Following the American Thoracic Society (ATS) guidelines,^[^ ^20^ ^]^ lung tissue and bronchoalveolar lavage (BAL) fluid were collected to assess tissue injury, alveolar‐capillary barrier alteration, and the inflammatory response. LPS challenge induced severe lung tissue injury in mice, characterized by neutrophil accumulation, alveolar wall thickening, and hyaline membrane formation (Figure 1B; Figure S1A, Supporting Information). These changes were markedly alleviated by GC‐1. Furthermore, GC‐1 significantly improved alveolar fluid clearance (AFC), increasing from 7.19% in the LPS group to 16.98% in the LPS+GC‐1 group (Figure 1C). Consistently, GC‐1 effectively reduced the lung wet/dry weight ratio and BAL fluid total protein concentration caused by LPS (Figure S1B,C, Supporting Information). Furthermore, GC‐1 decreased the number of LPS‐induced leukocytes and neutrophils in the BAL fluid (Figure 1D,E; Figure S1D,E, Supporting Information). ELISA findings confirmed increased levels of the proinflammatory cytokines IL‐1β and TNF‐α in mouse BAL fluid after LPS treatment, which were clearly decreased by GC‐1 administration (Figure 1F,G). In addition, we used another ALI model induced by HCl in mice to simulate gastric aspiration or lung/chest contusion in clinical cases, and the results showed that GC‐1 exhibited comparable efficacy in mitigating HCl‐induced pulmonary inflammation and tissue damage (Figure 1H,I; Figure S1F–L, Supporting Information). These findings suggest the promising potential of GC‐1 for the treatment of ALI/ARDS.

### 2.2 GC‐1 Selectively Inhibited the M1 Polarization of Macrophages

**Figure 2:**
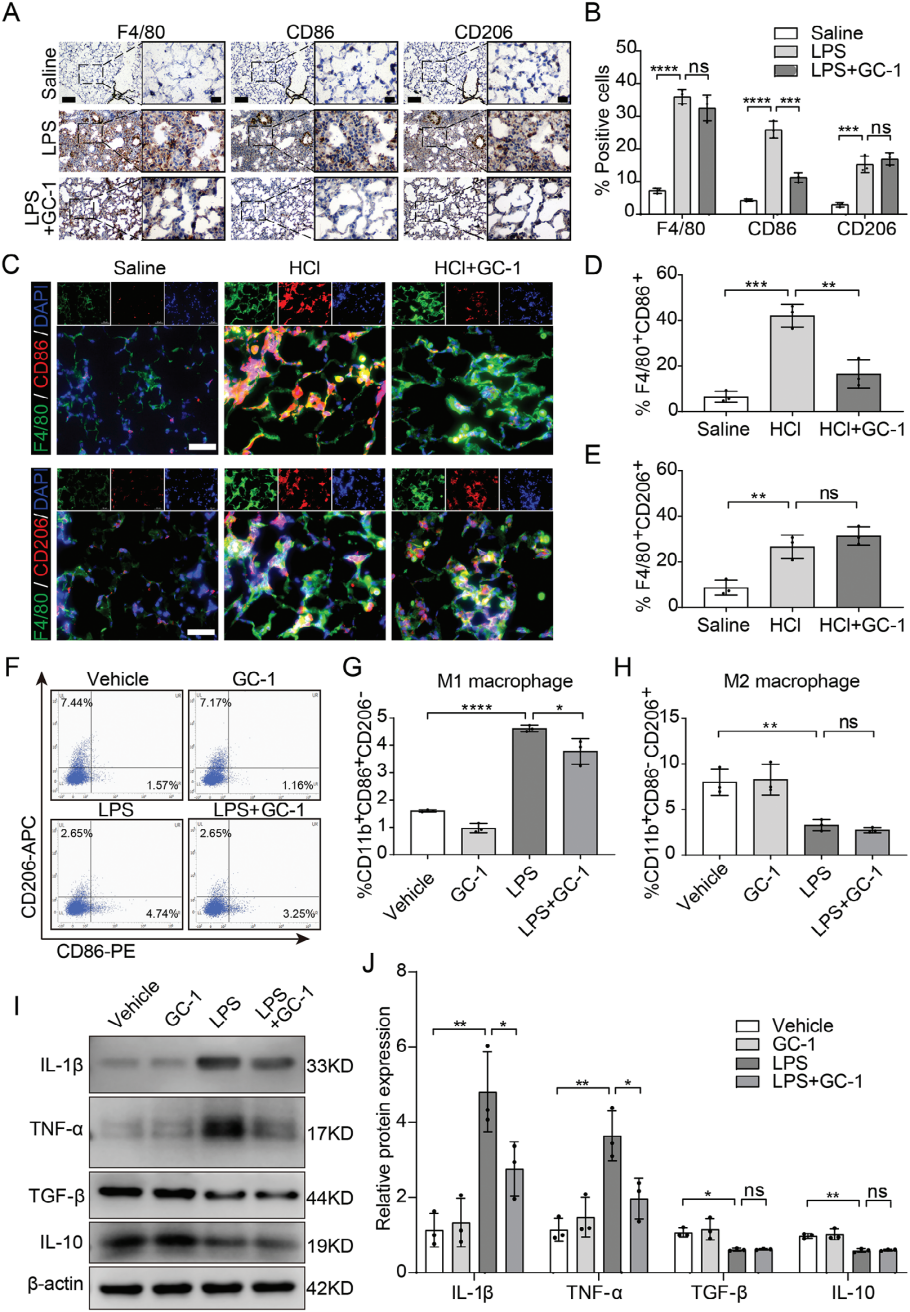
GC‐1 selectively inhibited the M1 macrophage polarization. A) Representative images of IHC staining for macrophage marker F4/80, M1 macrophage marker CD86, or M2 macrophage marker CD206 in lung sections of LPS‐induced ALI model mice (*n* = 3), Scale bars: 100 and 20 µm (insets). B) Quantitative statistical results of IHC‐stained positive cells (*n* = 3). C) Representative pictures of F4/80, CD86, and CD206 IF staining in lung sections from ALI mice induced by HCl (*n* = 3), Scale bars: 50 µm. Quantitative statistical results of fluorescence intensity from D) F4/80^+^CD86^+^ macrophages and E) F4/80^+^CD206^+^ macrophages (*n* = 3). F) Representative flow cytometry plots showing the proportions of M1 (CD11b^+^CD86^+^CD206^−^) and M2 (CD11b^+^CD86^−^CD206^+^) macrophages (*n* = 3). THP‐1 cells were treated with LPS (100 ng mL^−1^) alone or in combination with GC‐1 (100 nm) for 24 h after PMA induction. G) CD11b^+^CD86^+^CD206^−^ M1 macrophages and H) CD11b^+^CD86^−^CD206^+^ M2 macrophages quantitative statistical results (*n* = 3). I) Immunoblot analysis of lysates from THP‐1 cells (*n* = 3). J) Immunoblot gray values statistical quantification (*n* = 3). The values are shown as mean ± SD. ^*^ *p* < 0.05; ^**^ *p* < 0.01; ^***^ *p* < 0.001; ^****^ *p* < 0.0001; ns = not significant.

To investigate the impact of GC‐1 on macrophages in ALI, we performed immunohistochemical (IHC) staining on lung tissue sections from LPS‐induced ALI model mice. The results demonstrated a significant increase in F4/80, CD86, and CD206 expression in macrophages following LPS. However, GC‐1 effectively reduced CD86 expression without affecting F4/80 or CD206 levels (**Figure** **2**A,B). Similarly, the immunofluorescence (IF) staining of lung tissue from HCl‐induced ALI model mice confirmed the suppressive effect of GC‐1 on CD86 upregulation, while F4/80 and CD206 expression were not affected (Figure 2C–E). These findings suggested that GC‐1 selectively inhibited the M1 polarization of macrophages in ALI model mice without affecting M2 polarization. Next, we induced macrophage polarization in THP‐1 cells using LPS or IL‐4/13. Flow cytometry analysis revealed a significant reduction in the proportion of M1 (CD11b^+^CD86^+^CD206^−^) LPS‐induced THP‐1 macrophages upon treatment with GC‐1 but no effect on the proportion of macrophages with the M2 phenotype (CD11b^+^CD86^−^CD206^+^) (Figure 2F–H). Similarly, GC‐1 had no effect on the proportion of M2 IL‐4/13‐induced THP‐1 macrophages (Figure S2A–C, Supporting Information). WB and qRT‐PCR results for THP‐1 macrophages and murine primary AMs further confirmed that GC‐1 effectively suppressed the expression of the M1 phenotype markers IL‐1β, TNF‐α, and CD86 after LPS treatment but did not affect the M2 phenotype markers IL‐10, TGF‐β, or CD206 (Figure 2I,J; Figure S2D, Supporting Information). In conclusion, GC‐1 selectively inhibited M1 macrophage polarization without affecting M2 macrophage polarization.

### 2.3 GC‐1 Inhibited NF‐κB Activity to Immunomodulate M1 Macrophage Polarization

**Figure 3:**
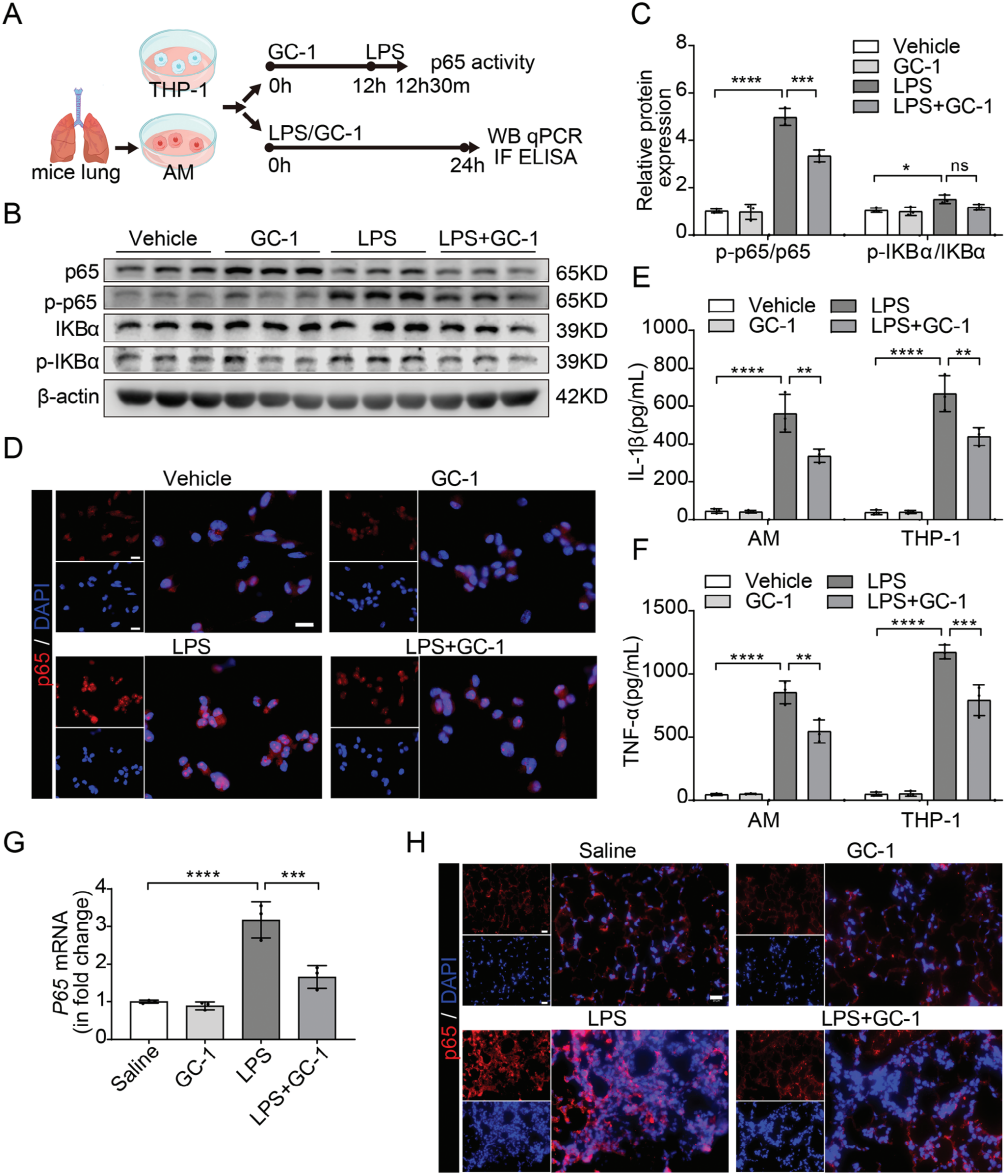
GC‐1 inhibited the LPS‐induced activation of the NF‐κB signaling. A) Schematic representation of the experiment. B) Western blot analysis was performed to assess the NF‐κB signaling activation in THP‐1 cells (*n* = 3). Prior to treatment with LPS (200 ng mL^−1^) for 30 min, THP‐1 cells were pretreated with 100 nM GC‐1 for 12 h. C) Immunoblot gray values statistical quantification (*n* = 3). D) Representative IF images of THP‐1 cells (*n* = 3), depicting the nuclear translocation of NF‐κB subunit p65 treated with LPS (200 ng mL^−1^) for 30 min, Scale bars: 20 µm. E) IL‐1β and F) TNF‐α levels in supernatants from AMs and THP‐1 cells treated with LPS (100 ng mL^−1^) and GC‐1 (100 nM) for 24 h were measured by ELISA (*n* = 3). G) qRT‐PCR analysis of p65 in murine lung tissue homogenate induced by LPS (*n* = 3). H) Representative pictures of p65 IF staining in lung sections from ALI model mice (*n* = 3), Scale bars: 20 µm. The values are shown as mean ± SD. ^*^ *p* < 0.05; ^**^ *p* < 0.01; ^***^ *p* < 0.001; ^****^ *p* < 0.0001; ns = not significant.

To investigate the mechanism of M1 polarization regulation in macrophages, we examined the impact of GC‐1 on NF‐κB signaling. THP‐1 macrophages were pretreated with GC‐1, followed by LPS stimulation for 30 min to assess changes in NF‐κB signaling activity (**Figure** **3**A). Immunoblot analysis revealed that LPS stimulation increased the phosphorylation levels of IKBα and p65 in THP‐1 cells, whereas GC‐1 effectively reduced p65 phosphorylation without inhibiting IKBα phosphorylation (Figure 3B,C). Moreover, LPS stimulation rapidly induced p65 nuclear import in THP‐1 cells, whereas GC‐1 markedly reduced the level of nuclear p65 (Figure 3D). These findings suggest that GC‐1 reduces p65 phosphorylation and nuclear translocation, thereby suppressing the increase in NF‐κB activity induced by LPS. The concentrations of proinflammatory factors in AM and THP‐1 cell supernatants confirmed the inhibitory effect of GC‐1 on the downstream target genes IL‐1β and TNF‐α (Figure 3E,F). Consistently, GC‐1 effectively suppressed p65 expression in ALI model mice induced by LPS (Figure 3G,H). Overall, our in vitro and in vivo experiments demonstrated that GC‐1 inhibited M1 macrophage polarization by attenuating NF‐κB activity.

### 2.4 The Suppression of NF‐κB Activity by GC‐1 Depended on PPARγ

**Figure 4:**
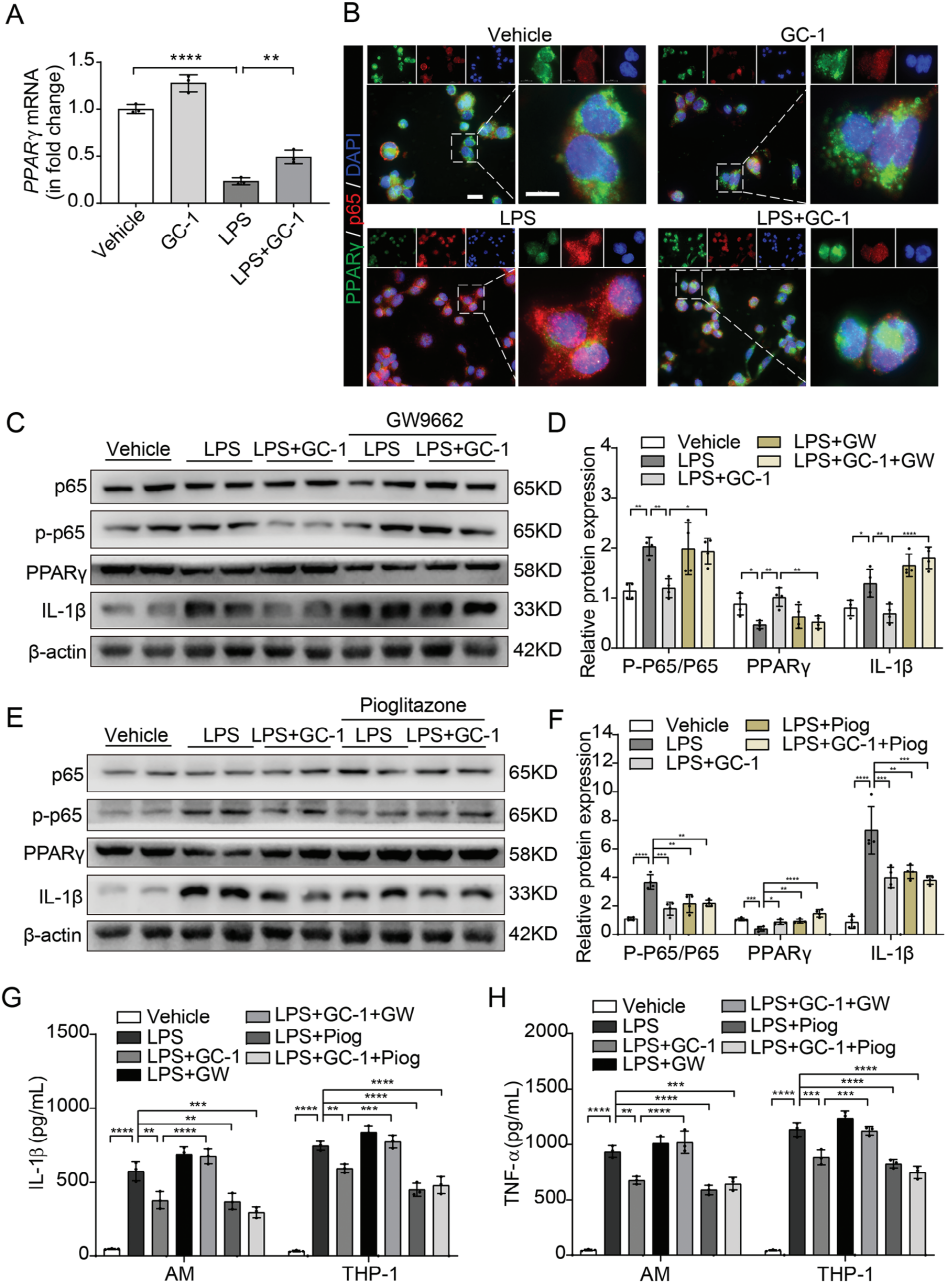
GC‐1 suppressed the activation of NF‐κB signaling in a PPARγ‐dependent manner. A) qRT‐PCR analysis of PPARγ mRNA expression in THP‐1 cells treated with LPS (100 ng mL^−1^) and GC‐1 (100 nm) for 24 h (*n* = 3). B) Representative images of P65 and PPARγ IF staining of THP‐1 cell slides treated with LPS (100 ng mL^−1^) and GC‐1 (100 nm) for 24 h (*n* = 3), Scale bars: 20 and 10 µm (insets). C) WB demonstrated phosphorylation of P65 in THP‐1 cells pretreated with GC‐1 (100 nm) along with the PPARγ inhibitor GW9662 (GW, 5 µm) for 12 h, followed by treatment with LPS (200 ng mL^−1^) for 30 min, as well as the expression of PPARγ and IL‐β in THP‐1 cells treated with LPS (100 ng mL^−1^), GC‐1 (100 nm) and GW9662 (5 µm) for 24 h. D) Immunoblot gray values statistical quantification (*n* = 4). E) WB analysis demonstrated the impact of the PPARγ agonist pioglitazone (Piog, 10 µm) on the phosphorylation of P65 and the expression levels of both PPARγ and IL‐1β in THP‐1 macrophage under similar treatment conditions mentioned above. F) Statistical quantification based on immunoblot gray values (*n* = 4). The levels of G) IL‐1β and H) TNF‐α in AM and THP‐1 cell supernatants were detected by ELISA (*n* = 3). The values are shown as mean ± SD. ^*^ *p* < 0.05; ^**^ *p* < 0.01; ^***^ *p* < 0.001; ^****^ *p* < 0.0001.

GC‐1 did not significantly inhibit IKBα phosphorylation induced by LPS, suggesting that GC‐1 may not regulate NF‐κB signaling through the classical pathway. Further analysis revealed that GC‐1 counteracted the inhibitory effects of LPS on PPARγ, indicating a potential mechanism by which GC‐1 may suppress NF‐κB activation through a PPARγ‐dependent pathway (**Figure** **4**A; Figure S3A–D, Supporting Information). Treatment with GC‐1 increased PPARγ expression and nuclear import while simultaneously reducing p65 expression induced by LPS, as shown by IF staining (Figure 4B). To explore the impact of GC‐1 on PPARγ, THP‐1 cells were treated with GW9662, a specific inhibitor of PPARγ, or pioglitazone, a specific agonist. The inhibitory effect of GC‐1 on LPS‐induced P65 phosphorylation was counteracted by GW9662, leading to the restoration of IL‐1β expression (Figure 4C,D). In contrast, pioglitazone mimicked GC‐1, effectively inhibiting LPS‐induced P65 phosphorylation and IL‐1β expression (Figure 4E,F). Additionally, GW9662 abrogated the GC‐1‐mediated inhibition of M1 proinflammatory factors induced by LPS in AM and THP‐1 cell supernatants (Figure 4G,H). These findings suggested that GC‐1 suppressed the M1 polarization of macrophages in a PPARγ‐dependent manner.

### 2.5 GC‐1 Increased PPARγ Expression by Blocking LPS‐Induced DNA Methyltransferase (DNMTs) 3b

**Figure 5:**
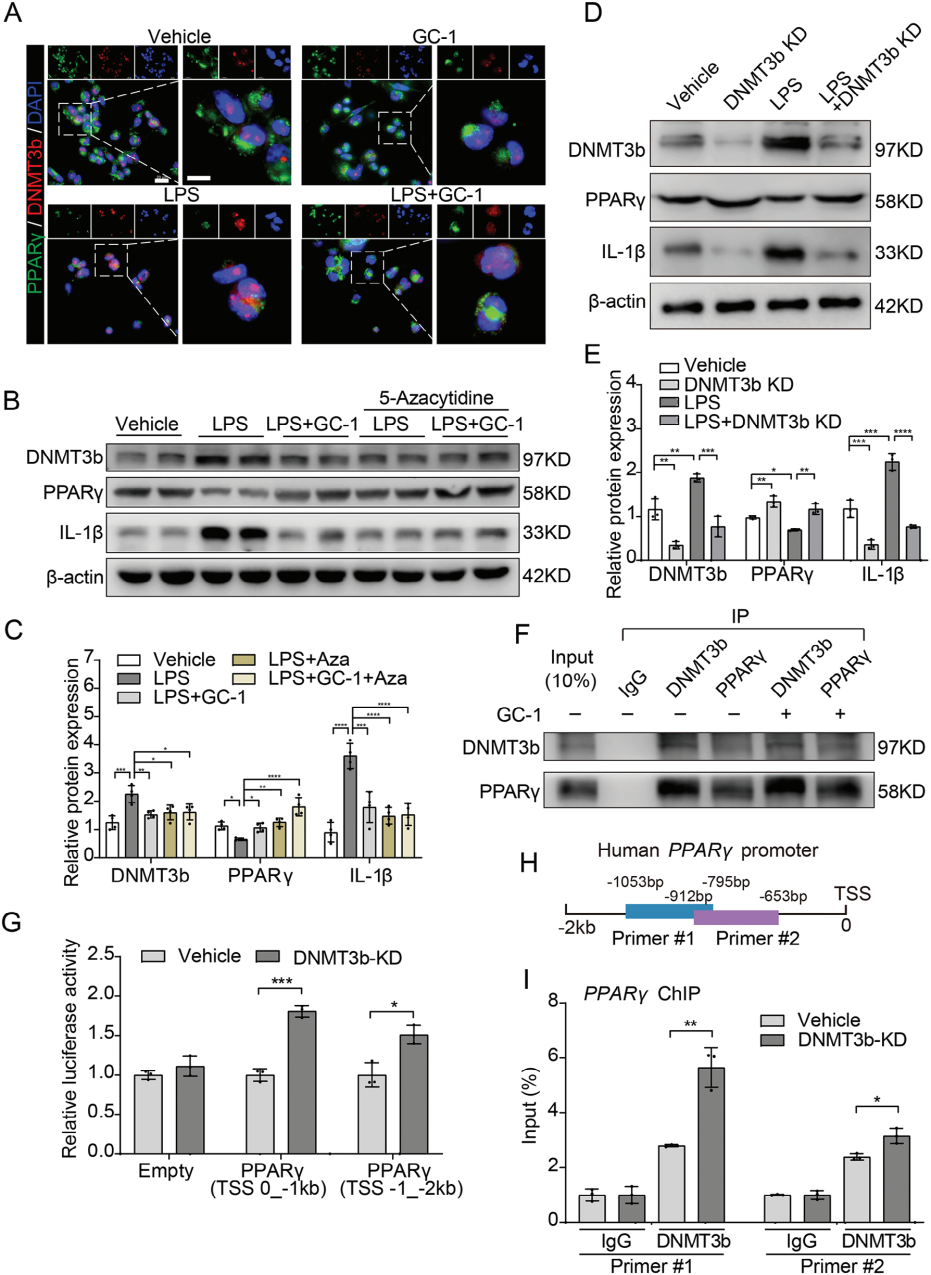
GC‐1 suppressed the upregulation of DNMT3b induced by LPS to increase the expression of PPARγ. A) Representative images of IF staining for DNMT3b and PPARγ in THP‐1 cells (*n* = 3), scale bars are 20 and 10 µm (insets). B) The level of protein expression of DNMT3b, PPARγ, and IL‐1β were analyzed by Western blotting in THP‐1 cells treated with the DNA methylation inhibitor 5‐azacytidine (Aza) (10 µm), LPS (100 ng mL^−1^), and GC‐1 (100 nm) for 24 h. C) Quantification of immunoblotting grayscale values (*n* = 4). D) Western blotting in THP‐1 cells was performed to investigate the impact of DNMT3b knockdown on PPARγ and IL‐1β production. E) Quantification of grayscale values in immunoblotting (*n* = 3). F) Endogenous Co‐IP experiment in THP‐1 cells to investigate the role of GC‐1 in the interaction of PPARγ and DNMT3b. G) The luciferase assays showed knocking down DNMT3b significantly increased PPARγ transcriptional activity in THP‐1 cells (*n* = 3). H) Schematic illustration of the primers used in the ChIP experiment, showing the position on the PPARγ promoter. I) ChIP assays demonstrated the binding of DNMT3b to the promoter region of PPARγ (*n* = 3). The values are shown as mean ± SD. ^*^ *p* < 0.05; ^**^ *p* < 0.01; ^***^ *p* < 0.001; ^****^ *p* < 0.0001; ns = not significant.

Previous studies have shown that PPARγ activity is epigenetically regulated by DNMTs. Thus, we investigated the impact of GC‐1 on the DNMT family. The upregulation of DNMT3b induced by LPS was effectively reduced by GC‐1, but there was no significant effect on the mRNA expression of DNMT1 and DNMT3a (Figure S4A–E, Supporting Information). These findings suggest a potential association between GC‐1 and DNMT3b in modulating PPARγ activity. GC‐1 reduced the DNMT3b expression and intranuclear accumulation induced by LPS, while simultaneously increasing PPARγ expression (**Figure** **5**A). Treatment of THP‐1 cells with 5‐azacytidine, a specific inhibitor of DNA methylation, effectively blocked the induction of DNMT3b by LPS. Additionally, it resulted in an increase in PPARγ expression and a reduction in IL‐1β expression (Figure 5B,C). 5‐Azacytidine also decreased the levels of proinflammatory cytokines induced by LPS in AM and THP‐1 cells (Figure S4F,G, Supporting Information). In line with the effects of 5‐azacytidine, knockdown of DNMT3b effectively counteracted the inhibitory impact of LPS on PPARγ and attenuated IL‐1β production (Figure 5D,E). ELISA results from THP‐1 cell supernatants also confirmed that silencing DNMT3b effectively attenuated the production of the LPS‐induced proinflammatory factors IL‐1β and TNF‐α (Figure S4H, Supporting Information). Moreover, the results obtained from an endogenous Co‐IP experiment in THP‐1 cells demonstrated a clear interaction between PPARγ and DNMT3b, while the impact of GC‐1 on this interaction appeared to be negligible (Figure 5F). This finding suggested that GC‐1 may not directly influence the interplay between these two proteins. The dual luciferase reporter and ChIP assays provided evidence suggesting that DNMT3b exhibited binding affinity for the PPARγ promoter region and that DNMT3b knockdown significantly increased PPARγ transcriptional activity (Figure 5G–I).

### 2.6 LPS‐Induced Activation of DNMT3b Was Inhibited by GC‐1 Via TRβ1

**Figure 6:**
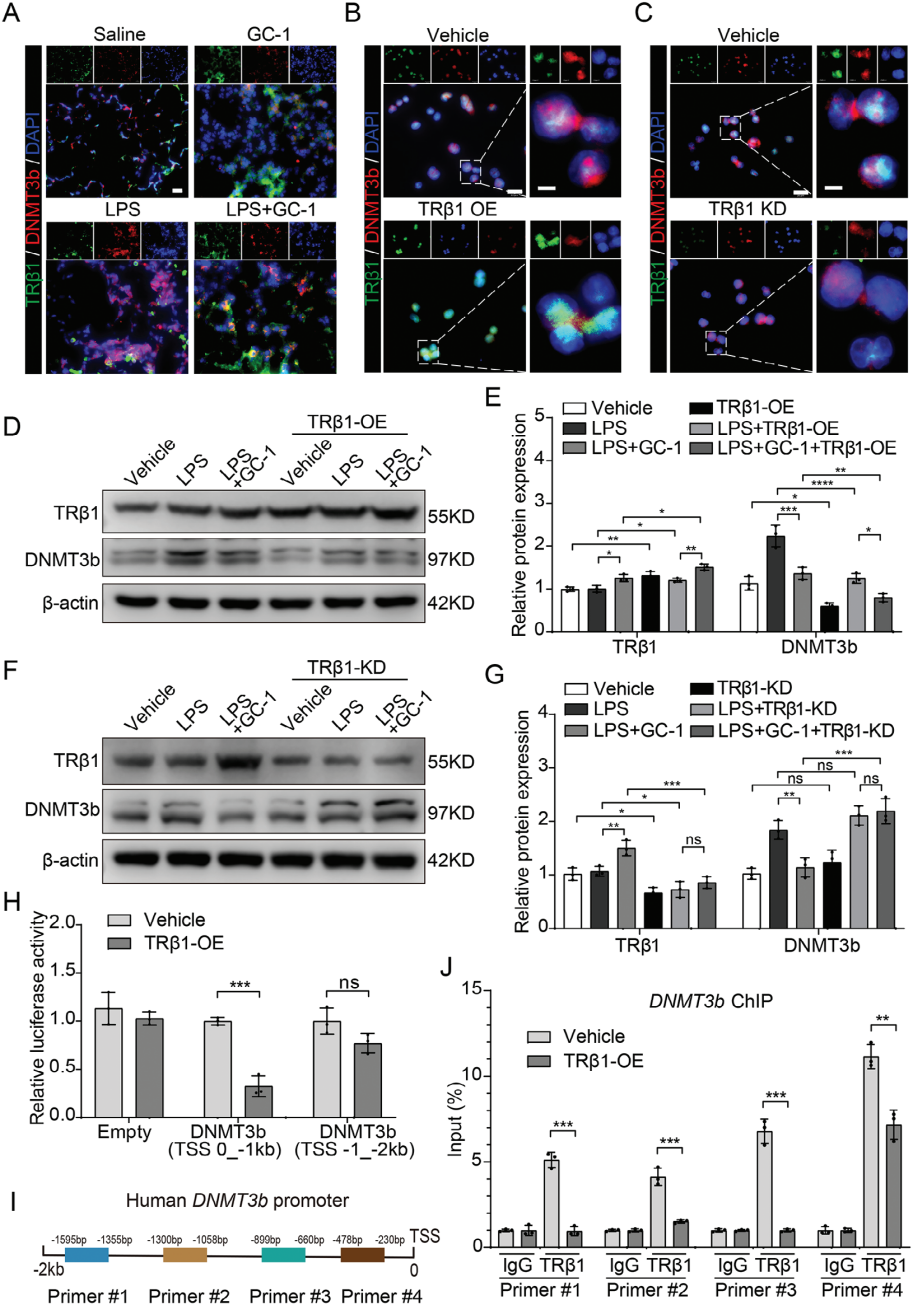
GC‐1 inhibited the activation of DNMT3b induced by LPS via TRβ1. A) Representative images of IF staining for DNMT3b and TRβ1 in LPS‐induced ALI model mice (*n* = 3), scale bar is 20 µm. Illustrative images of IF staining for B) TRβ1 overexpression (OE) and C) TRβ1 knockdown (KD) in THP‐1 cells (*n* = 3), scale bars are 20 and 5 µm (insets). D) The impact of TRβ1 overexpression on DNMT3b production was investigated in THP‐1 cells using Western blotting. E) Gray values statistical quantification (*n* = 3). F) Western blotting in THP‐1 cells to investigate the impact of TRβ1 knockdown on DNMT3b production. G) Quantification of grayscale values in immunoblotting (*n* = 3). H) The luciferase assays demonstrated TRβ1 overexpression significantly attenuated DNMT3b transcriptional activity in THP‐1 cells (*n* = 3). I) Schematic illustration of the primers used in the ChIP, indicating their position on the DNMT3b promoter. J) ChIP assays provided evidence of TRβ1 binding to DNMT3b promoter (*n* = 3). The values are shown as mean ± SD. ^*^ *p* < 0.05; ^**^ *p* < 0.01; ^***^ *p* < 0.001; ^****^ *p* < 0.0001; ns = not significant.

The TH analog GC‐1 selectively binds to TRβ1. Therefore, knockdown and overexpression experiments targeting TRβ1 were performed to elucidate whether TRβ1 influences DNMT3b expression. The IF results demonstrated that treatment with GC‐1 or TRβ1 overexpression significantly suppressed the expression of DNMT3b. Moreover, TRβ1 and DNMT3b were colocalized (**Figure** **6**A,B). However, knockdown of TRβ1 seemed to have no effect on the expression of DNMT3b in THP‐1 cells (Figure 6C). Furthermore, TRβ1 overexpression markedly attenuated the upregulation of DNMT3b caused by LPS and enhanced the inhibitory effect of GC‐1 (Figure 6D,E). Conversely, TRβ1 knockdown abolished the inhibitory effect of GC‐1 on LPS‐induced DNMT3b (Figure 6F,G). This suggested that TRβ1 may have an inhibitory effect on DNMT3b, while GC‐1 was inhibited through this receptor‐dependent mechanism. The dual‐luciferase reporter and ChIP results further confirmed that TRβ1 can bind to the promoter region of DNMT3b and that the overexpression of TRβ1 significantly reduced the transcriptional activity of DNMT3b (Figure 6H–J).

### 2.7 Inhibition of DNMT3b Attenuated M1 Macrophage Polarization and Lung Injury in ALI Model Mice

**Figure 7:**
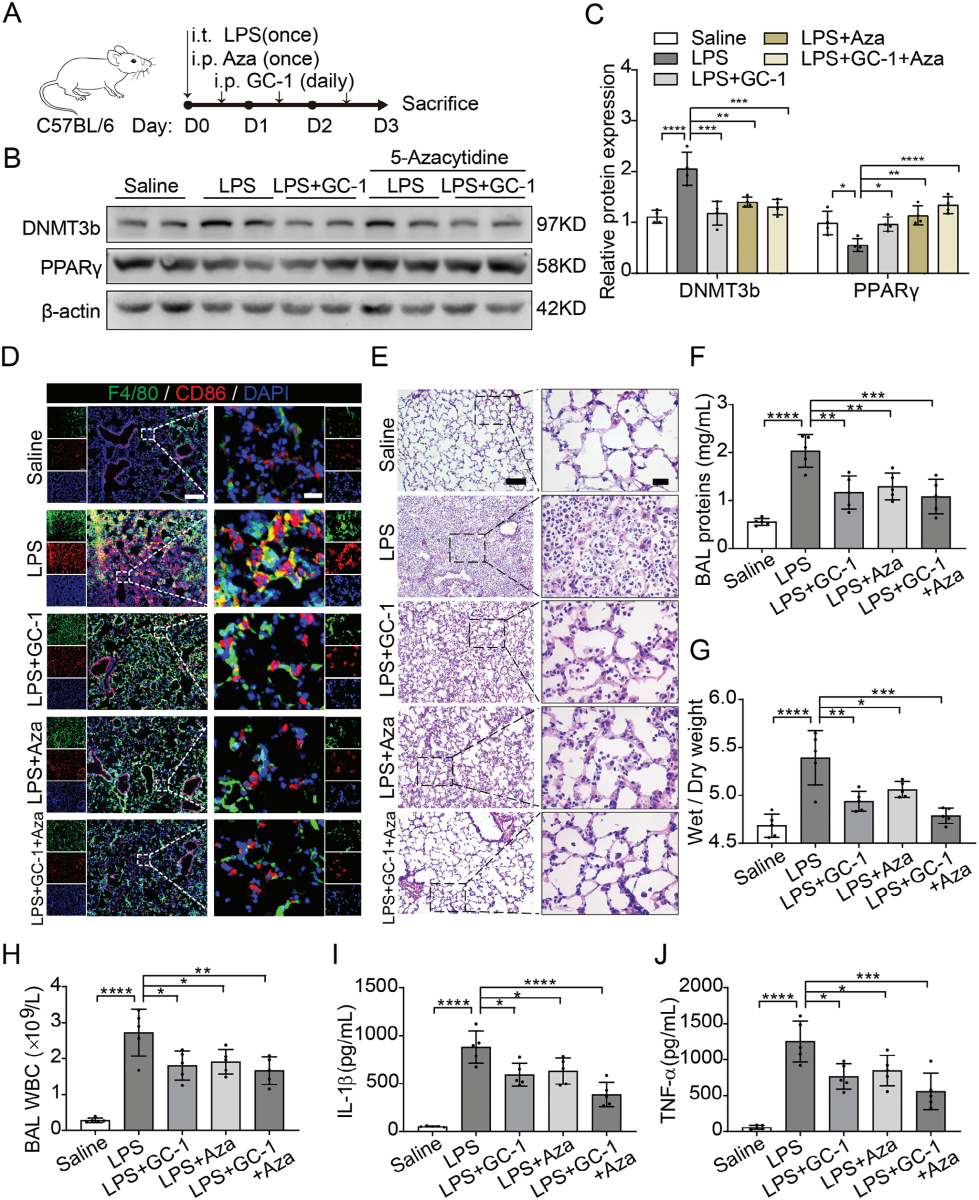
Inhibition of DNMT3b restrained M1 macrophage polarization and lung injury in ALI model mice. A) Schematic representation of the experiment. After treatment with LPS, C57BL/6 mice were intraperitoneally injected with DNA methylation inhibitor 5‐azacytidine (Aza) (1 mg kg^−1^ body weight), while other treatment methods are mentioned in Figure 1. B) WB analysis of the effects of Aza on the expression of DNMT3b, PPARγ, and IL‐1β in mouse lungs. C) Quantification results for immunoblotting grayscale values (*n* = 4). D) IF staining for F4/80 and CD86 in lung slices from mice (*n* = 3). Scale bars: 200 and 20 µm(insets). E) Representative histological sections of lung stained with H&E (*n* = 3). Scale bars: 100 and 20 µm (insets). Lung injury was evaluated by measuring F) the BAL fluid total protein content, G) lung wet/dry weight ratio, and H) BAL fluid WBC concentration (*n* = 5). The concentrations of I) IL‐1β and J) TNF‐α in mouse BAL fluid were determined using ELISA assays (*n* = 5). The values are shown as mean ± SD. ^*^ *p* < 0.05; ^**^ *p* < 0.01; ^***^ *p* < 0.001; ^****^ *p* < 0.0001.

To validate the in vitro results, we administered intraperitoneal injections of GC‐1 and/or 5‐azacytidine to LPS‐induced ALI model mice (**Figure** **7**A). Lung tissue homogenates were analyzed by immunoblotting, which revealed that, compared with LPS alone, both GC‐1 and 5‐azacytidine effectively reduced DNMT3b levels and increased PPARγ expression (Figure 7B,C). IF staining of lung tissue confirmed these findings (Figure S5A, Supporting Information). Furthermore, both GC‐1 and 5‐azacytidine inhibited M1 macrophage polarization (F4/80^+^CD86^+^) induced by LPS in mice, as evidenced by the significant suppression of CD86 expression and its colocalization with F4/80 (Figure 7D). Lung tissue pathology and BAL fluid analysis revealed that GC‐1 and 5‐azacytidine alleviated lung tissue damage and alveolar‐capillary barrier injury caused by LPS. The combined administration of GC‐1 and 5‐azacytidine resulted in superior therapeutic effects (Figure 7E–H). Additionally, GC‐1 and 5‐azacytidine decreased TNF‐α and IL‐1β levels in BAL fluid triggered by LPS (Figure 7I,J). These findings suggest that GC‐1 and 5‐azacytidine may hinder M1 macrophage polarization, alleviating lung injury and inflammation in ALI model mice.

## 3 Discussion

In this study, the ability of GC‐1 to treat ALI was evaluated in two murine models. Our results showed that GC‐1 reduced pulmonary damage caused by LPS or HCl, including thickened alveolar walls, disrupted capillary barriers, and neutrophil infiltration. It also lowered inflammatory cytokine levels, improving disease progression. Further studies revealed that GC‐1 may inhibit NF‐κB p65 signaling through the DNMT3b‐PPARγ‐NF‐κB pathway in a TRβ1‐dependent manner, reducing M1 macrophage polarization and inflammatory cytokine production. These findings suggest that GC‐1 could be a promising anti‐inflammatory drug with immunomodulatory properties for ALI/ARDS and inflammation‐related diseases.

THs have been found to regulate metabolism, growth, and development.^[^ ^8^ ^]^ Recently, their potential in modulating M1 macrophages in ALI has gained attention.^[^ ^21^ ^]^ This study revealed that GC‐1 effectively reduced the number of overactivated M1 macrophages in ALI model mice, which complemented our previous research on the ability of GC‐1 to restore mitochondrial function and reduce lung epithelial cell injury and fibrosis induced by bleomycin and hyperoxia.^[^ ^16^ ^]^ The shared origin of the lung and thyroid during embryonic development has sparked scientific interest in the regulatory role of THs and thyromimetics in lung development and pulmonary diseases despite the controversial history of TH therapy.^[^ ^14^, ^22^ ^]^ THs play crucial roles in various human diseases, including fatty liver disease, heart failure, kidney disease, severe acute respiratory syndrome (SARS) and COVID‐19.^[^ ^23^ ^]^ Our results suggest that GC‐1 may also play novel roles in lung injury repair. Phase I clinical trials have demonstrated the promising efficacy and safety of GC‐1.^[^ ^11^ ^]^ As an endogenous hormone analog, GC‐1 is expected to be safer than other exogenous medications. Further research on the interaction between hormones and the immune system in treating lung diseases shows promise for clinical practice.

The COVID‐19 outbreak has highlighted the importance of precision medicine for the personalized treatment of ALI/ARDS.^[^ ^3^ ^]^ We used two murine models to mimic ALI and found dysregulated control of macrophage polarization, neutrophil infiltration, and proinflammatory cytokines in both models. GC‐1 inhibited M1 macrophage polarization by suppressing NF‐κB signaling, reducing lung damage and inflammation in mice. These findings suggest that GC‐1 could be a potential therapy for ALI caused by NF‐κB‐mediated macrophage polarization. Studies have emphasized the importance of macrophage polarization in ALI/ARDS development,^[^ ^24^ ^]^ while inhibiting NF‐κB activation effectively reduces the inflammatory response and lung damage in ALI/ARDS.^[^ ^25^ ^]^ In conclusion, targeting the NF‐κB p65 pathway in macrophage polarization may constitute a focused and effective therapeutic approach.

Research on macrophage polarization therapy for ALI/ARDS can be classified into three groups: strategies that target M1 macrophages, M2 macrophages, or a combination of both. Precision medicine requires the implementation of targeted therapeutic strategies on the basis of specific features during each stage of the disease.^[^ ^26^ ^]^ During the exudative phase of ALI/ARDS, excessive inflammation and infiltration of inflammatory cells are caused primarily by hyperactivation of M1 macrophages.^[^ ^27^ ^]^ Targeting M1 macrophages may be an effective approach in this phase. Treatment with GC‐1 effectively inhibited excessive M1 macrophage polarization, reducing inflammation and neutrophil infiltration. These findings suggest that GC‐1 has therapeutic potential in the exudative phase of ALI/ARDS. Notably, GC‐1 selectively inhibited M1 macrophage polarization without affecting M2 macrophage polarization, which was associated with fibrosis in advanced stages of ALI/ARDS.^[^ ^27^, ^28^ ^]^ Building upon our previous discovery of the alleviating effect of GC‐1 on pulmonary fibrosis in mice,^[^ ^16^, ^29^ ^]^ it can be inferred that this specific immunomodulation seems to be more conducive to the antifibrotic effect of GC‐1. The results of injecting stem cells in ALI treatment support this inference.^[^ ^30^ ^]^ It effectively inhibits M2 macrophage polarization and reduces collagen deposition in mice with ALI. The unique regulatory mechanism of GC‐1 may contribute to its dual anti‐inflammatory and antifibrotic functions in lung injury. This may be a valuable reference for designing future precision medicine drugs.

Macrophage polarization is a highly regulated process involving complex signaling pathways, transcriptional epigenetics, and posttranscriptional regulation.^[^ ^17^ ^]^ Our findings revealed that GC‐1 inhibited the phosphorylation and nuclear translocation of P65 induced by LPS through PPARγ‐NF‐κB signaling, thereby suppressing M1 macrophage polarization. The PPARγ inhibitor GW9662 reversed the GC‐1‐mediated inhibition of LPS‐induced NF‐κB signaling and TNF‐α and IL‐1β expression. Conversely, the PPARγ agonist pioglitazone enhanced the effect of GC‐1. These results strongly support the involvement of PPARγ in mediating the inhibitory effect of GC‐1 on LPS‐induced NF‐κB signaling. Previous studies have shown that PPARγ inhibits M1 macrophage activation by blocking the expression of M1 marker genes through antagonizing AP‐1, STAT, and NF‐κB activities.^[^ ^31^ ^]^ Our findings align with those of Gellrich et al.,^[^ ^32^ ^]^ who demonstrated that TH activates PPARγ signaling by binding to its ligand‐binding domain. These results emphasize the potential of targeting PPARγ with GC‐1 to explore the therapeutic benefits of THs and thyromimetics.

Studies have shown that elevated DNMT1/3a or DNMT3b levels in patients with idiopathic pulmonary fibrosis and obesity can cause DNA methylation of the PPARγ promoter, thus suppressing PPARγ expression.^[^ ^33^ ^]^ Our findings revealed increased DNMT3b and decreased PPARγ after LPS stimulation. Treatment with GC‐1 and 5‐azacytidine effectively counteracted the LPS‐induced inhibition of PPARγ by decreasing DNMT3b, thereby suppressing NF‐κB signaling activation and M1 polarization of macrophages. These results suggest that GC‐1 may promote the PPARγ signaling through the DNMT3b‐PPARγ pathway. In a study on the mouse brain, Kyono et al. reported that Dnmt3a may function as a T_3_ response gene and identified two functional T_3_ response elements located 30.3 and 49.3 kb from the transcription start site of Dnmt3a. Deletion of the 30.3 kb TRE eliminated or strongly reduced the Dnmt3a mRNA response to T_3_. Further bioinformatics analysis revealed that both TREs are highly conserved among eutherian mammals.^[^ ^34^ ^]^ In a mouse brain injury model, short‐term administration of T_3_ reduced the expression levels of DNMT3a/3b and de novo DNA methylation.^[^ ^35^ ^]^ These findings suggest that thyroid hormones and GC‐1 regulate the expression of DNMT families by binding to their receptors, indicating their potential epigenetic regulatory function in stress responses such as ALI/ARDS, in addition to their known roles as developmental epigenetic and transgenerational epigenetic modifiers.^[^ ^36^ ^]^

## 4 Conclusion

The current study demonstrated that the TH analog GC‐1 exhibited selective immunomodulatory effects on M1 macrophage polarization in ALI and is a promising drug candidate for ALI/ARDS and inflammation‐related diseases. Mechanistically, GC‐1 may reduce p65 phosphorylation and nuclear translocation, thereby inhibiting NF‐κB signaling activation through the DNMT3b‐PPARγ‐NF‐κB pathway, which is dependent on TRβ1. Moreover, this research advances the understanding of the role of THs and thyromimetics in immune regulation associated with lung inflammation and repair, as well as revealing the interaction between the endocrine and immune systems. This exploration has promising implications for clinical practice and personalized medicine in patients with endocrine and pulmonary disorders.

## 5 Experimental Section

### Animals

The 8‐week‐old male C57BL/6 mice were purchased from Beijing Charles River Laboratory Animal Technology Co., Ltd. (Beijing, China) and housed in a specific pathogen‐free environment. They were randomly divided into four groups for ALI induction. On day 0, a single dose of 5 mg kg^−1^ LPS (O111:B4, Sigma–Aldrich, Missouri, USA) dissolved in 50 µL saline, or 50 µL HCl (0.1 m, pH 1.0) was administered intratracheally as previously described.^[^ ^16a^ ^]^ Control mice received saline. After 12 h, the mice were treated with an intraperitoneal injection of 100 µg kg^−1^ GC‐1 (Sigma–Aldrich) dissolved in 0.2 mL saline or saline daily for three days. Mice were given an intraperitoneal injection of 1 mg kg^−1^ 5‐azacytidine (HY‐10586, MCE, Shanghai, China) in 0.2 mL saline after LPS administration. The care and handling procedures for animals followed the guidelines set by Henan Normal University Institutional Animal Care and Use Committee (IACUC SMKX‐2118BS1018), adhering to standards established by the Animal Behavior Society and National Regulations.

### Cell Culture

The THP‐1 cell lines were purchased from Procell (CL‐0233, Pricella Life Science&Technology Co.,Ltd, Wuhan, China) and cultured in RPMI‐1640 medium (PM150110, Procell) supplemented with 10% fetal bovine serum (v/v), 100 U mL^−1^ penicillin, and 100 mg L^−1^ streptomycin (HY‐K1006, MCE). The cells were maintained at 37 °C with 5% CO_2_. Cell lines were tested for Mycoplasma using Myco‐Lumi Luminescent Mycoplasma Detection Kit (C0298S, Beyotime, Shanghai, China). LPS was dissolved in PBS while GC‐1, phorbol 12‐myristate 13‐acetate (PMA) (HY‐18739, MCE), 5‐azacytidine (HY‐10586, MCE), GW9662 (HY‐16578, MCE), and pioglitazone (HY‐13956, MCE) were dissolved in DMSO. Mouse primary alveolar macrophages were isolated using BAL. After removing red blood cells, the cellular pellet was suspended in RPMI‐1640 medium and incubated at 37 °C with 5% CO_2_ for 4 to 6 h before changing the medium. The adherent cells were identified as macrophages using immunofluorescence and Diff‐Quik staining methods.

### Alveolar Fluid Clearance

After intraperitoneal anesthesia, a 20 G (1.1 mm) intravenous needle was inserted into the upper part of the trachea. Evans Blue (0.15 mg mL^−1^, HY‐B1102, MCE) labeled 5% albumin solution was slowly infused through the catheter in increments of 50 µL every 2 min until a total volume of 300 µL. Concurrently, pure oxygen inhalation was provided (4 mL min^−1^). After 20 min, mice were sacrificed and fluid absorbance at 620 nm was measured to calculate AFC (%).

### BAL Fluid White Blood Cells (WBC) Concentration

In this experiment, an automated hematology analyzer (DxH 500, Beckman Coulter, California, USA) was used for the detection of WBC concentration retrieved from mouse BAL fluid. Briefly, after collection, the BAL fluid was chilled and 0.2 mL was extracted and mixed with an appropriate volume of normal saline to ensure a suitable detection range. Subsequently, following the manufacturer's instructions, the analysis was conducted and the results were documented.

### BAL fluid Cell Smear

Mouse BAL fluid was centrifuged and the cell precipitate was resuspended in saline before being transferred to a centrifuge funnel (Thermo Scientific) placed on a slide spinner (Cytospin4, Thermo Scientific). Slides were then stained and the neutrophil count was determined by examining three random fields with at least 200 cells each.

### qRT‐PCR

Total RNA was extracted using the miRNeasy Mini kit (QIAGEN, Dusseldorf, Germany). Subsequently, RNA reverse transcription was performed using Prime Script Reverse Transcriptase (Promega, Wisconsin, USA). qRT‐PCR was conducted with qPCR Master Mix (Promega) according to the manufacturer's instructions. β‐actin served as an internal reference. The primer pairs utilized in this study are presented in **Table** **1**.

**Table 1:** qRT‐PCR primers in this study.

Primer name	Sequence (5′−3′)
ACTB‐F	CATGTACGTTGCTATCCAGGC
ACTB‐R	CTCCTTAATGTCACGCACGAT
DNMT1‐F	AGGTGGAGAGTTATGACGAGGC
DNMT1‐R	GGTAGAATGCCTGATGGTCTGC
DNMT3a‐F	GTCATGTGGTTCGGAGACGG
DNMT3a‐R	AGTGTCACTCTCATCGCTGTC
DNMT3b‐F	TAACAACGGCAAAGACCGAGGG
DNMT3b‐R	TCCTGCCACAAGACAAACAGCC
PPARa‐F	ATGGTGGACACGGAAAGCC
PPARa‐R	CGATGGATTGCGAAATCTCTTGG
PPARg‐F	ACCAAAGTGCAATCAAAGTGGA
PPARg‐R	ATGAGGGAGTTGGAAGGCTCT
PPARd‐F	ACTGAGTTCGCCAAGAGCATC
PPARd‐R	ACGCCATACTTGAGAAGGGTAA
mACTB‐F	GGCTGTATTCCCCTCCATCG
mACTB‐R	CCAGTTGGTAACAATGCCATGT
mp65‐F	TGCGATTCCGCTATAAATGCG
mp65‐R	ACAAGTTCATGTGGATGAGGC
mCD86‐F	TCAATGGGACTGCATATCTGCC
mCD86‐R	GCCAAAATACTACCAGCTCACT
mCD206‐F	CTCTGTTCAGCTATTGGACGC
mCD206‐R	TGGCACTCCCAAACATAATTTGA
mIL1B‐F	GAAATGCCACCTTTTGACAGTG
mIL1B‐R	TGGATGCTCTCATCAGGACAG
mTNF‐F	CAGGCGGTGCCTATGTCTC
mTNF‐R	CGATCACCCCGAAGTTCAGTAG
mIL10‐F	GCTGGACAACATACTGCTAACC
mIL10‐R	ATTTCCGATAAGGCTTGGCAA
mArg1‐F	CTCCAAGCCAAAGTCCTTAGAG
mArg1‐R	GGAGCTGTCATTAGGGACATCA

### Western Blot

Protein samples (20 µg) were separated by SDS‐PAGE gels and transferred to PVDF membranes (Millipore, Massachusetts, USA). After blocking, the membranes were incubated overnight at 4 °C with specific primary antibodies: anti‐β‐actin (T0022, Affinity), anti‐IL10 (DF6894, Affinity), anti‐TGF‐β (BF8012, Affinity), anti‐TNF‐α (AF7014, Affinity), anti‐p65 (AF5006, Affinity), anti‐Phospho p65 (AF2006, Affinity), anti‐IKBα (AF5002, Affinity), anti‐Phospho IKBα (AF2002, Affinity), anti‐PPARγ (16643‐1‐AP, Proteintech), anti‐IL‐1β (AF5103, Affinity), anti‐DNMT3b (26971‐1‐AP, Proteintech), anti‐TRβ1 (sc‐738, Santa Cruz). Then they were incubated for 2 h with HRP‐labeled secondary antibodies. Blots were visualized using an enhanced chemiluminescence system (Bio‐Rad, USA) with β‐actin as a reference.

### Immunohistochemistry

Experimental procedures followed IHC kit instructions (Beyotime). Lung tissue sections were incubated overnight at 4 °C with primary antibodies against CD86 (DF6332, Affinity), CD206 (DF4149, Affinity), and F4/80 (34 028 M, Bioss), then photographed using a light microscope.

### Hematoxylin and Eosin Staining

After deparaffinization and rehydration, mouse lung tissue sections were stained with hematoxylin for nuclear labeling and eosin for cytoplasmic staining (Beyotime). The sections were then dehydrated, sealed, and photographed using light microscopy.

### Immunofuorescence

The frozen lung tissue sections were fixed with 4% paraformaldehyde and treated with 0.3% Triton X‐100 (HY‐Y1883A, MCE) as previously described.^[^ ^37^ ^]^ Primary antibodies including anti‐PPARγ, anti‐DNMT3b, anti‐CD86, anti‐CD206, and anti‐F4/80 were incubated overnight at 4 °C. Fluorescent secondary antibodies were then incubated in darkness for an hour. Finally, the nuclei were labeled using DAPI, and images were acquired with a Zeiss fluorescence microscope.

### ELISA

TNF‐α and IL‐1β levels in mouse lung tissue, BAL fluid, and cell supernatant were measured using ELISA kit (SEKM‐0034, SEKH‐0047, SEKM‐0002, SEKH‐0002, Solarbio Science & Technology, Beijing, China) as instructed.

### Flow Cytometry

According to the instructions, APC Anti‐Human CD206 Antibody (E‐AB‐F1161E, Elabscience Biotechnology), PE Anti‐Human CD86 Antibody (E‐AB‐F1012D, Elabscience Biotechnology), and FITC Anti‐Human CD11b Antibody (E‐AB‐F1081C, Elabscience Biotechnology) were separately added.

### Small Interfering RNA (siRNA) Knockdown

Follow the instructions, THP‐1 cells were transfected with siRNA genOFF h‐DNMT3b (SIGS0002749‐1, Ribobio, Guangzhou, China), or siRNA genOFF h‐THRB (SIGS0000943‐1, Ribobio) to knockdown DNMT3b or TRβ1 using Lipofectamine 3000 (L3000015, ThermoFisher).

### Chromatin Immunoprecipitation Combined with Quantitative PCR

THP‐1 cells were cross‐linked with 1% formaldehyde for 10 min at RT. Fixation was stopped by 0.25 m glycine for 5 min and the samples were washed twice with ice‐cold PBS. Cell pellets were then resuspended in 1 mL of cyto lysis buffer, mixed briefly, and incubated on ice for 10–15 min with occasional inversion every 2 min. Cells were then centrifuged for 5 min at 3500 rpm at 4 °C, the supernatant was discarded, and the remaining nuclear pellet (white) was resuspended in 500 µL of nuclear lysis buffer (1% SDS, 10 mm EDTA, 50 mM Tris‐Cl, pH 8.1, 1× protease inhibitor cocktail (HY‐K0010, MCE)). The samples were then sonicated seven times at the maximum setting (BRANSON SLPe, output setting 4, 10 sec per cycle). The soluble chromatin was collected by centrifuging for 10 min at 14000 rpm, and the supernatant was diluted 1:10 with dilution buffer (150 mm NaCl, 20 mm Tris‐HCl, pH 8.1, 2 mm EDTA, 1% Triton X‐100 and 1× protease inhibitor cocktail). Chromatin was incubated at 4 °C overnight with protein A/G beads that pre‐coupled with anti‐DNMT3b (26971‐1‐AP, Proteintech) and anti‐TRβ1 antibody (sc‐738, Santa Cruz). The complexes with beads were washed twice in low‐salt buffer (150 mm NaCl, 20 mm Tris‐HCl, pH 8.1, 2 mm EDTA, 1% Triton X‐100, 0.1% SDS), twice in high‐salt buffer (500 mm NaCl, 20 mm Tris‐HCl, pH 8.1, 2 mm EDTA, 1% Triton X‐100, 0.1% SDS), twice in LiCl buffer (250 mm LiCl, 1% NP‐40, 1% deoxycholate, 1 mm EDTA, pH8.0, 10 mm Tris‐HCl, pH 8.1) and twice in TE buffer (pH 8.0). The thoroughly washed beads were eluted twice with 150 µL of elution buffer (0.1 m NaHCO_3_, 1% SDS) by vortexing at 70 °C at 1000 rpm for 10 min on a Thermo Mixer C (Eppendorf). The enriched DNA fragments were then purified with Qiaquick spin column and quantified by Nanodrop. The published primers of PPARγ and DNMT3b were used for DNMT3b and TRβ1 occupancy analysis with SYBR green master mix (17747200, Roche). Primers used for ChIP–qPCR are listed in **Table** **2**.

**Table 2:** ChIP–qPCR primers in this study.

Primer name	Sequence (5′−3′)
DNMT3b‐1‐F	AGAAAGCCAAGGAGTCCTGC
DNMT3b‐1‐R	GCTCCAGCTCCCTGAGTTAC
DNMT3b‐2‐F	TTGATGCATCCCCCATCCAC
DNMT3b‐2‐R	GGCTTTTCCCGATGACTCCA
DNMT3b‐3‐F	AGGCAGAGAGGGGTTAAGGT
DNMT3b‐3‐R	GCCCTGGGATGACCATTGAA
DNMT3b‐4‐F	TCCAAAGCAGGATGACAGGC
DNMT3b‐4‐R	TGGGGGATCAGAAGCCCTAA
PPARg‐1‐F	CAGGTCAGAGTACGGGTGC
PPARg‐1‐R	CTCAGCCCAAGACCCCTTC
PPARg‐2‐F	GATGAGAGCTGGGGAGAAGG
PPARg‐2‐R	TCCTGGAAGCCGGGACG

### Co‐Immunoprecipitation

For immunoprecipitations, THP‐1 cells were lysed with NP40 lysis buffer (10 mm Tris‐Cl, pH 7.4, 100 mm NaCl, 2.5 mm MgCl_2_, 0.5% NP 40) supplemented with 1 mm PMSF and 1× proteinase inhibitor cocktail (P8340, Sigma) on ice for 15 min, and then briefly sonicated three times with BRANSON SLPe (output setting 4, 10 s per cycle). After spinning the lysed cells at 13000 rpm for 20 min at 4 °C, the supernatant was transferred to a new eppendorf tube. Pre‐cleared with 10 µL of protein A/G magnetic beads (88803, Pierce) for 1 h at 4 °C; then the lysate was incubated with 10 µg of either anti‐DNMT3b or anti‐PPARγ antibody and rotated overnight at 4 °C. The samples were then incubated with 50 µL of protein A/G magnetic beads for 4 h at 4 °C. After thoroughly washing with NP40 lysis buffer for five times, DNMT3b or PPARγ enriched proteins were eluted with 1× LDS sample buffer for 10 min at 70 °C by vortexing at 1000 rpm with a Thermo Mixer C. The eluted proteins were then analyzed by Western blotting.

### Luciferase Reporter Assay

For the luciferase reporter assay, oligonucleotides containing the transcription start site sequence of DNMT3b/PPARγ were amplified and cloned into a pGL3 vector immediately upstream of the luciferase gene. To identify the influence of DNMT3b on PPARγ or TRβ1 on DNMT3b activity, DNMT3b and PPARγ reporter were constructed. HEK293 cells were transfected with the luciferase constructs and siRNA using Lipofectamine 3000. Luciferase activity was measured at 48 h post‐transfection with a Dual‐Luciferase assay system (Cat No.11402ES60, Yeasen, Shanghai, China) according to the manufacturer's protocols. Primers used for luciferase reporter assay are listed in **Table** **3**.

**Table 3:** Primers for luciferase reporter assay in this study.

Primer name	Sequence (5′−3′)
DNMT3b‐1‐F (TSS −1_−2 kb)	TGGTGGCTCATGCTTGTAATCCCAGCACTTT
DNMT3b‐1‐R (TSS −1_−2 kb)	TTTGCAGGACCAGATTCTTACCCCTCGGTTTT
DNMT3b‐2‐F (TSS 0_−1 kb)	GATCTGGGCAAGAAATGTCCAGGTGTA
DNMT3b‐2‐R (TSS 0_−1 kb)	TGGGGAGGGGGCGGTGCCGACTCCCCTT
PPARg‐1‐F (TSS −1_−2 kb)	GACTGGAAAAAGGGAAAGAGGGGCCTCAAGT
PPARg‐1‐R (TSS −1_−2 kb)	GCCCAGCCAGCAGCCGGTTCCCGAG
PPARg‐2‐F (TSS 0_−1 kb)	GGGGAGTGCTCAGGGAGGGGGCGC
PPARg‐2‐R (TSS 0_−1 kb)	CCCTTCCTTACCTACCACTGAC

### Statistical Analyses

The data were analyzed using GraphPad Prism 7.0 (GraphPad Software, Inc. Serial: GPS‐0320559‐LFUL‐95242). For comparison among multiple groups, the one‐way ANOVA with Tukey's multiple comparison test was employed. For two groups, an unpaired Student's *t*‐test was used. All data were presented as mean ± standard deviation (SD). *p* < 0.05 was considered statistically significant.

## Conflict of Interest

The authors declare no conflict of interest.

## Supporting information

Supporting Information

## Data Availability

Data sharing is not applicable to this article as no new data were created or analyzed in this study.
