# Supplementary material for: The Thyroid Hormone Analog GC‐1 Mitigates Acute Lung Injury by Inhibiting M1 Macrophage Polarization
Source: Adv Sci (Weinh). 2024 Oct 7;11(44):2401931. doi: 10.1002/advs.202401931 (PMC11600256; doi:10.1002/advs.202401931)
Supplement: Supplementary file 1 — Supporting Information [file ADVS-11-2401931-s001.docx]

Supporting Information

**Title**

**The Thyroid Hormone Analog GC-1 Mitigates** **Acute Lung Injury by** **Inhibiting** **M1 Macrophage Polarization**

**Authors**

*Bin Li, Cong Xia, Wanyu He, Jingyi Liu, Ruoyu Duan, Zhihua Ji, Xiaoyue Pan, Yanlin Zhou, Lan Wang^*^, Guoying Yu^*^*

**
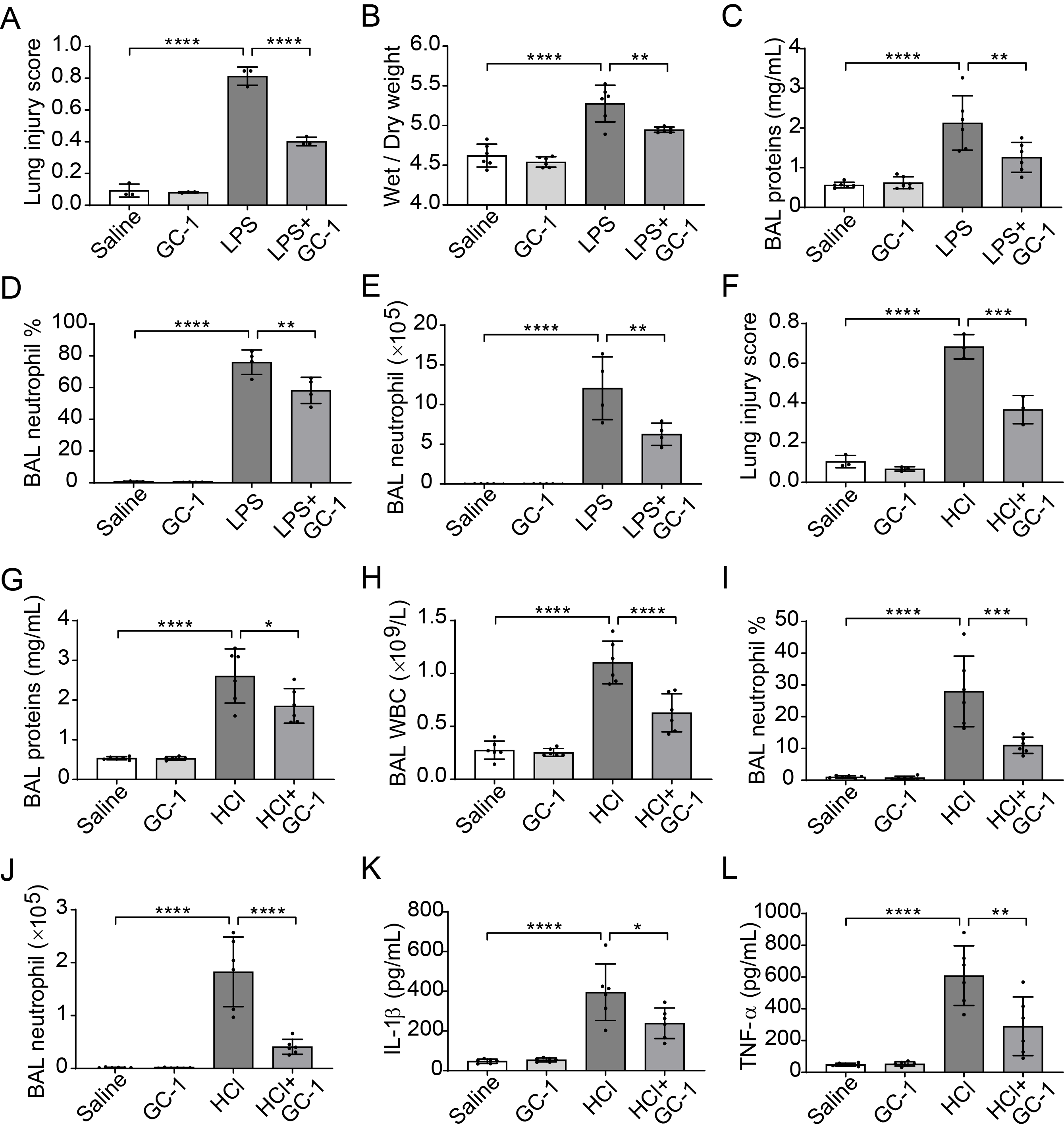
**

**Figure S1.** **GC-1 attenuated lung injury in mice with ALI induced by LPS or HCl.** (**A**) Lung injury score of LPS-induced ALI according to the ATS lung injury scoring system (n= 3). LPS-induced lung injury was evaluated by (**B**) lung wet/dry weight ratio (n= 6), and (**C**) BAL fluid total protein levels (n= 6). Three random fields per slide were examined to calculate LPS-induced (**D**) BAL fluid neutrophil percentage and (**E**) total neutrophil count (n= 4). (**F**) Lung injury score of HCl-induced ALI (n= 3). HCl-induced lung injury was evaluated by (**G**) BAL protein levels (n= 6), (**H**) WBC concentration in BAL (n= 6), (**I**) the percentage of neutrophils in BAL fluid (n= 6), as well as (**J**) the number of neutrophils (n= 6). ELISA was used to measure (**K**) IL-1β and (**L**) TNF-α levels in HCl-induced BAL fluid (n= 6). The values are shown as mean ± SD. **P* < 0.05; ***P* <0.01; ****P* < 0.001; *****P* < 0.0001.

**
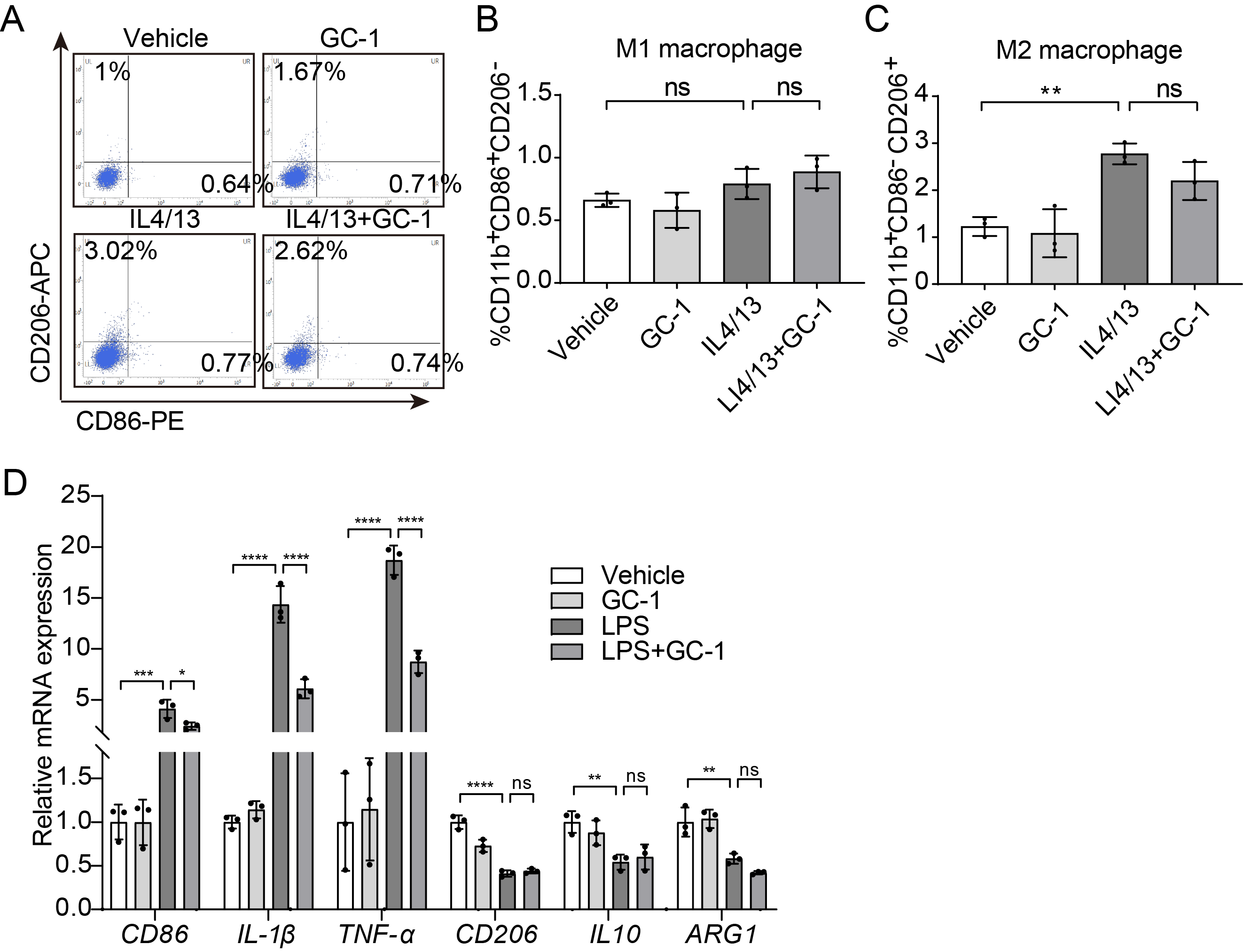
**

**Figure S2. GC-1 did not affect M2 macrophage polarization.** (**A**) Flow cytometry plots illustrating the percentage of M1 (CD11b^+^CD86^+^CD206^-^) and M2 (CD11b^+^CD86^-^CD206^+^) macrophages (n= 3). THP-1 cells were subjected to PMA treatment for 24 hours followed by IL4 (20ng/mL) combined with IL13 (20ng/mL) treatment, then treated with GC-1 (100nM) for 24 hours. Quantification of the proportion of (**B**) CD11b^+^CD86^+^CD206^-^ M1 macrophages and (**C**) CD11b^+^CD86^-^CD206^+^ M2 macrophages (n= 3). (**D**) qRT-PCR analysis of macrophage markers in mouse AMs treated with LPS (100ng/mL) and GC-1 (100nM) for 24 hours (n= 3). The values are shown as mean ± SD. **P* < 0.05; ***P* <0.01; ****P* < 0.001; *****P* < 0.0001; ns = not significant.

**
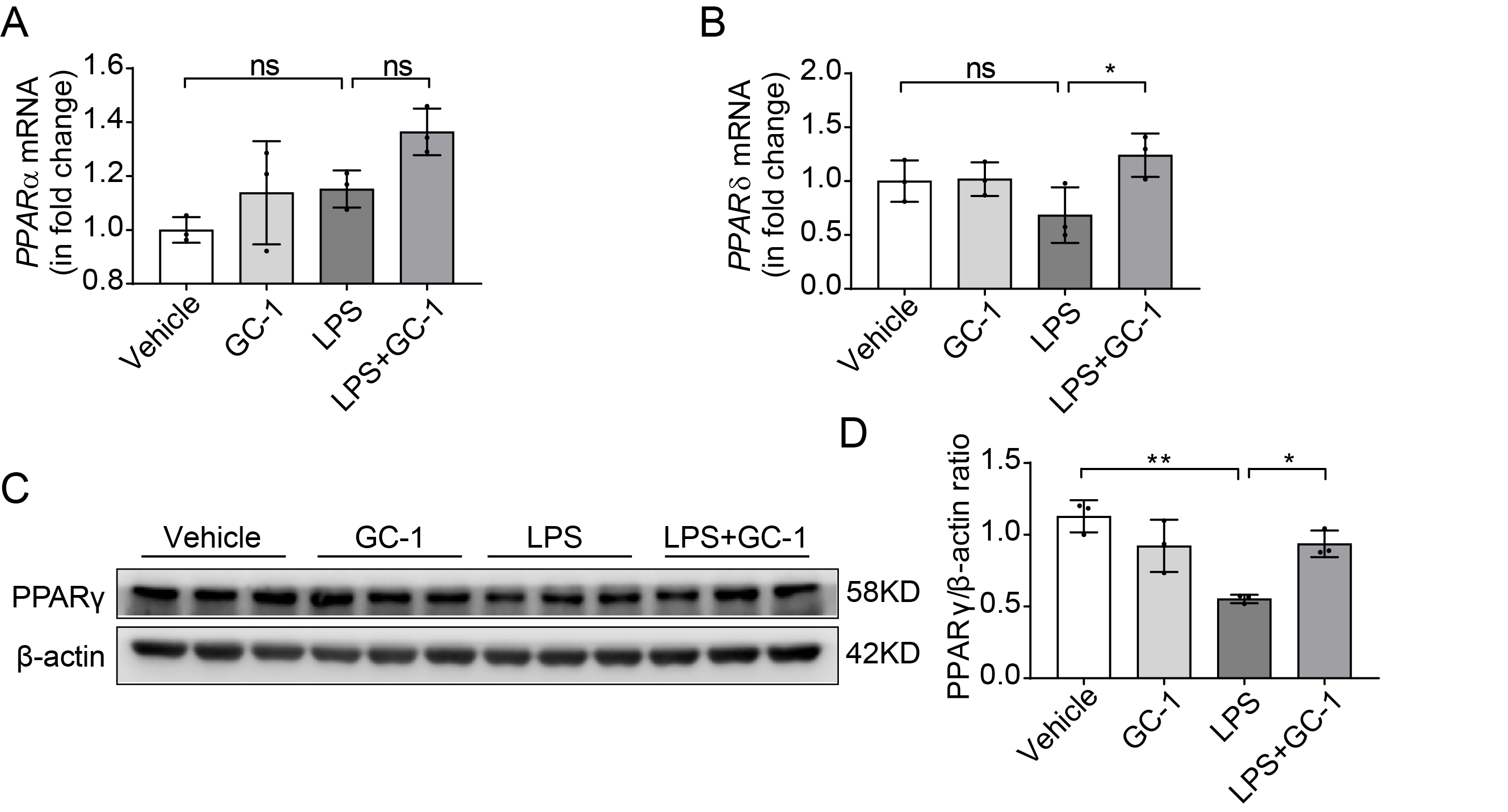
**

**Figure S3.** **The inhibitory effect of LPS on PPARγ in THP-1 cells was abolished by GC-1.** qRT-PCR analysis of (**A**) *PPARα* mRNA, (**B**) *PPARδ* mRNA and WB analysis of (**C**) PPARγ protein expression in THP-1 cells treated with LPS (100ng/mL) and GC-1 (100nM) for 24 hours (n= 3). (**D**) a statistical analysis of immunoblot gray values (n = 3). The values are shown as mean ± SD. **P* < 0.05; ***P* <0.01; ns = not significant.

**
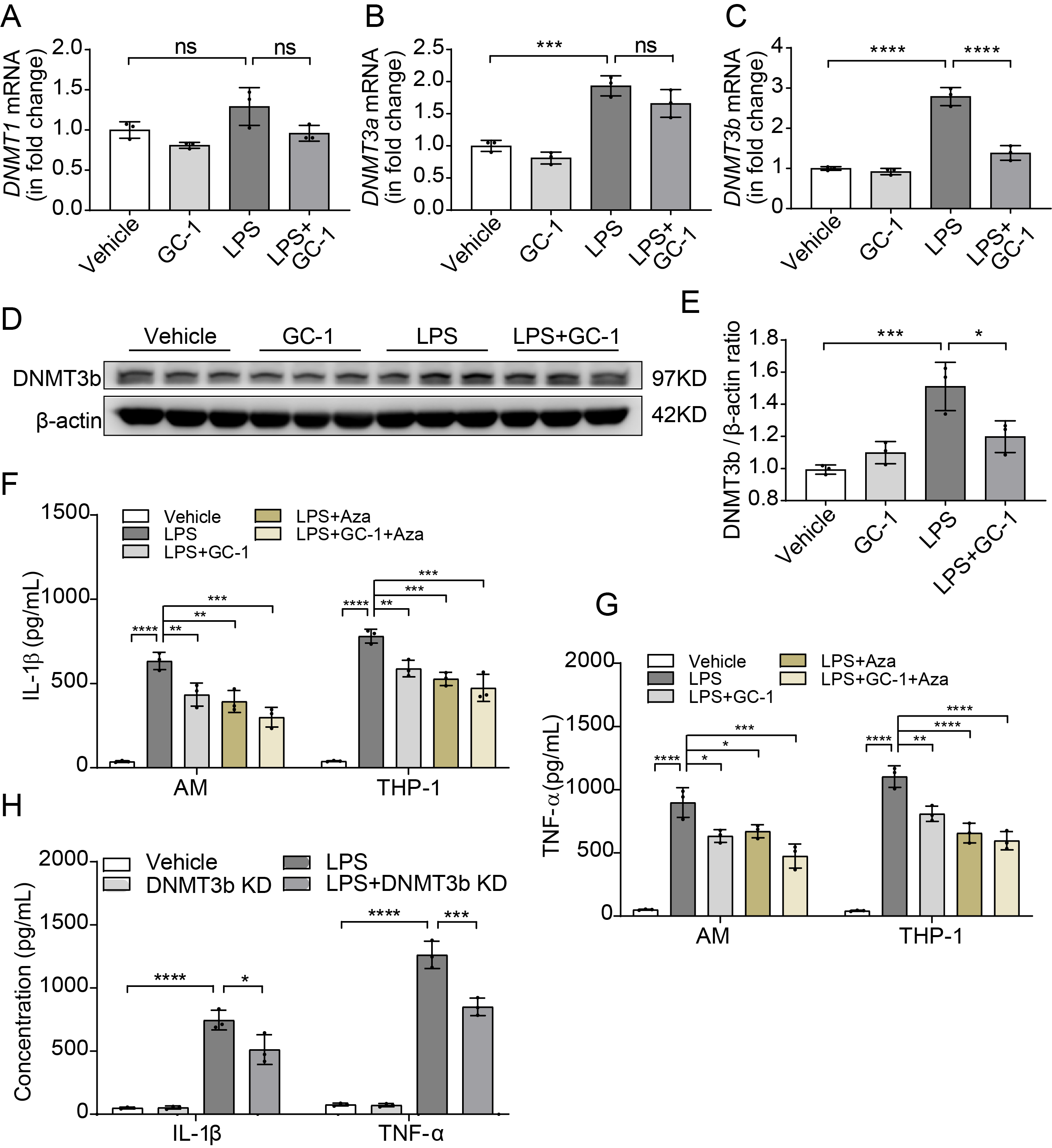
**

**Figure S4. GC-1 inhibited the LPS-induced DNMT3b, thereby promoting the expression of PPARγ.**

qRT-PCR analysis was conducted to assess the mRNA expression levels of (**A**) *DNMT1,* (**B**) *DNMT3a* and (**C**) *DNMT3b* in THP-1 cells treated with LPS (100ng/mL) and GC-1 (100nM) for 24 hours (n= 3). WB analysis of (**D**) DNMT3b protein expression in THP-1 cells treated with LPS (100ng/mL) and GC-1(100nM) for 24 hours (n= 3). (**E**) a statistical analysis of immunoblot gray values (n= 3). The levels of (**F**) IL-1β and (**G**) TNF-α in the supernatant of AM and THP-1 cells were detected by ELISA (n= 3). (**H**) The levels of IL-1β and TNF-α in THP-1 cell supernatant were measured using ELISA (n= 3). The values are shown as mean ± SD. **P* < 0.05; ***P* <0.01; ****P* < 0.001; *****P* < 0.0001; ns = not significant.

**
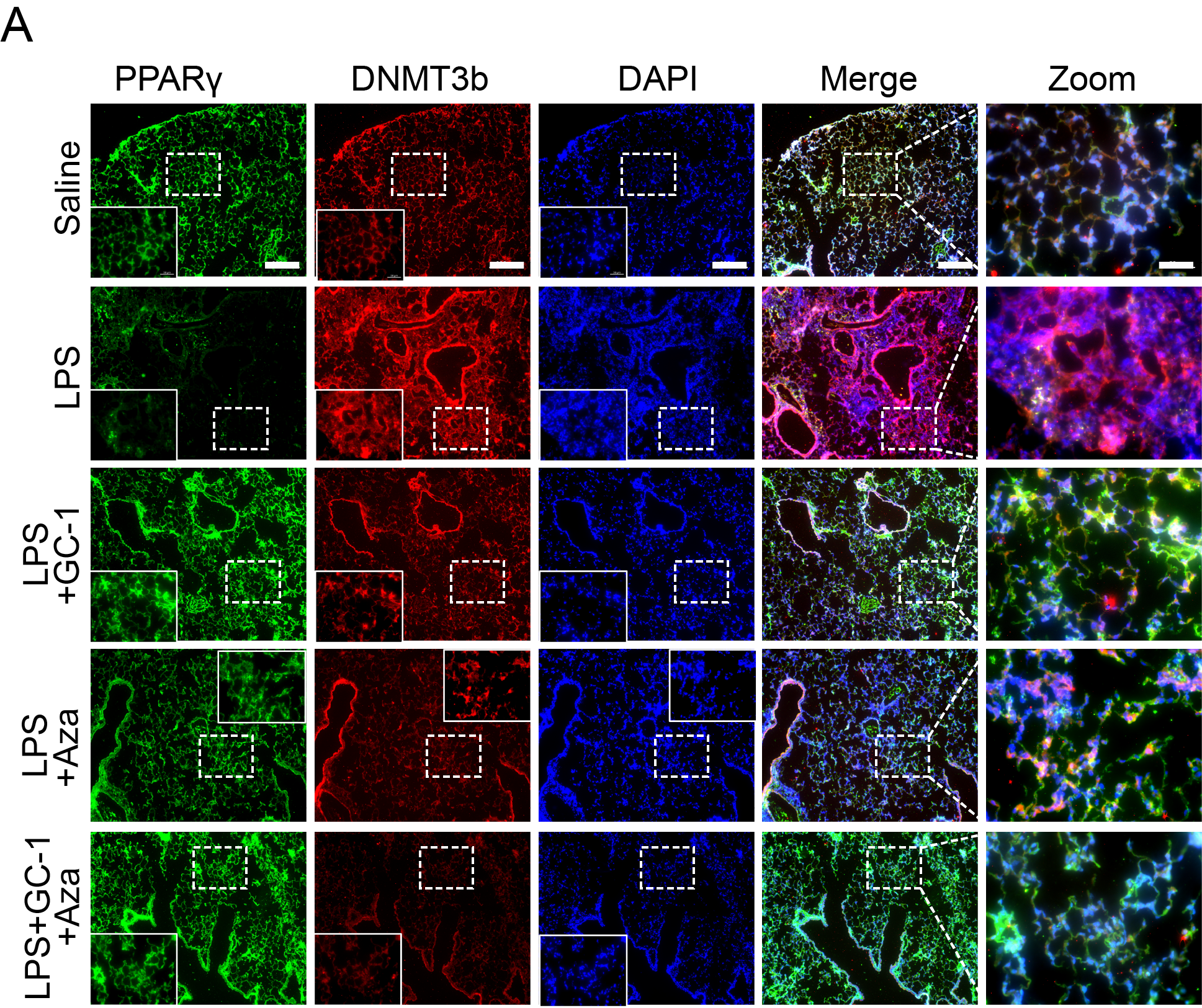
**

**Figure S5. Effects of GC-1 and 5-azacytidine on the expression of DNMT3b and PPARγ in LPS-induced ALI model mice.** (**A**) Representative images showing IF staining of DNMT3b and PPARγ in mouse lung sections (n= 3), Scale bars: 200 µm and 50 µm (insets).
